# A Glimmer of Hope: Recent Updates and Future Challenges in Zika Vaccine Development

**DOI:** 10.3390/v12121371

**Published:** 2020-11-30

**Authors:** Priscila M. S. Castanha, Ernesto T. A. Marques

**Affiliations:** Graduate School of Public Health, Department of Infectious Diseases and Microbiology, University of Pittsburgh, Pittsburgh, PA 15261, USA; pmd35@pitt.edu

**Keywords:** Zika virus, vaccine candidates, vaccine platforms, clinical trials

## Abstract

The emergence and rapid spread of Zika virus (ZIKV) on a global scale as well as the establishment of a causal link between Zika infection and congenital syndrome and neurological disorders triggered unprecedented efforts towards the development of a safe and effective Zika vaccine. Multiple vaccine platforms, including purified inactivated virus, nucleic acid vaccines, live-attenuated vaccines, and viral-vectored vaccines, have advanced to human clinical trials. In this review, we discuss the recent advances in the field of Zika vaccine development and the challenges for future clinical efficacy trials. We provide a brief overview on Zika vaccine platforms in the pipeline before summarizing the vaccine candidates in clinical trials, with a focus on recent, promising results from vaccine candidates that completed phase I trials. Despite low levels of transmission during recent years, ZIKV has become endemic in the Americas and the potential of large Zika outbreaks remains real. It is important for vaccine developers to continue developing their Zika vaccines, so that a potential vaccine is ready for deployment and clinical efficacy trials when the next ZIKV outbreak occurs.

## 1. Introduction

Zika virus (ZIKV), a member of the *Flaviviridae* family (*Flavivirus* genus), is a positive, single-stranded, enveloped RNA virus primarily transmitted to humans through the bite of infected *Aedes aegypti* mosquitoes [1,2]. ZIKV was initially isolated in 1947 as part of routine surveillance investigations of the yellow fever transmission cycle carried out by the Rockefeller Institute in the Zika forest of Uganda [1]. During the 50 years following its discovery, only few sporadic Zika cases associated with mild, self-limiting febrile disease were detected in humans in Africa and in some Asian countries [3,4,5]. The first large outbreak of Zika disease in humans was documented in 2007 on the Pacific Island of Yap, Federated States of Micronesia [6]. Seven years later, additional ZIKV outbreaks in humans were also registered on other Pacific Islands, including French Polynesia [7,8,9]. In early 2015, a large outbreak of Zika disease was registered in Brazil [10,11,12] and ZIKV spread rapidly throughout the Americas [13,14]. Until then, ZIKV disease was considered a benign viral infection with minor health consequences, but a causal link between ZIKV infection and clusters of microcephaly and neurological disorders was later recognized [15,16,17], leading the WHO to declare the ZIKV outbreak as a Public Health Emergency of International Concern in February 2016 [18].

Much has been learned during the ZIKV outbreak in the Americas. Multiple, previously unknown routes of human-to-human ZIKV transmission have been identified, including via sexual [19,20,21,22,23,24], intrauterine, and intrapartum transmission [16,17,25,26,27,28,29,30,31], and via blood transfusion [8,32,33]. Evidence from epidemiological studies has also shown that ZIKV infection sporadically triggers Guillain–Barré syndrome, a rare but serious autoimmune-mediated attack of healthy peripheral neurons and glial cells, leading to ascending paralysis and polyneuropathy and a small number of cases of confirmed viral encephalitis. Cases of ZIKV-associated Guillain–Barré syndrome have been reported in Brazil, Colombia, French Polynesia, and several other countries [34,35,36,37,38,39,40]. However, perhaps the most dramatic ZIKV feature discovered during the Americas outbreak has been the impact of ZIKV infections on pregnant women and their infants. Fetal microcephaly linked to Zika infections during pregnancy was first reported in Brazil [16,17,29,31]. Later, Zika-related microcephaly was also identified in other South and Central American countries, and in retrospective analyses of the French Polynesia outbreak [41,42,43,44,45,46]. Other Zika-associated congenital disorders have also been identified and include intrauterine growth restriction, fetal demise, cerebral calcifications, sensorineural hearing loss, visual impairment [26,28,30,47,48,49,50], and delayed cognitive development. However, the full spectrum of clinical presentations of congenital Zika syndrome is yet to be described.

Intense research efforts on the underlying pathogenic mechanisms of Zika infections have also been made during the 2015–2016 Zika outbreak. Within a relatively short period of time, models of Zika disease and pathogenesis in mice and non-human primates have been established, including using female pregnant animals (reviewed in [51]). These studies have revealed ZIKV tropism to maternal and fetal tissues, including a broad range of placental cell types and the developing fetal human brain [25,26,27,52,53,54]. Prolonged persistence of ZIKV genome in several body tissues and fluids, including the male and female reproductive tract, has also been identified [25,55,56]. The close antigenic relationship that results in considerable serological cross-reactivity between ZIKV and other flaviviruses, particularly dengue viruses (DENV), has also been a topic of intense research. The fact that poorly neutralizing cross-reactive antibodies can potentially mediate antibody-dependent enhancement (ADE) of Zika or dengue infections has been extensively explored using in vitro and ex vivo experimental approaches, animal models, and epidemiological analyses [57,58,59,60,61,62,63,64,65]. Antibody responses to DENV have been shown to be highly cross-reactive to ZIKV and vice-versa. In fact, several analyses have shown that these Zika and dengue cross-reactive antibodies are able to promote either enhancement of or protection against subsequent flavivirus infections, an effect that seems to depend on the antibody levels at the time of infection [57,58,59]. However, it is still unknown how these Zika and dengue antibody interactions will impact future vaccination efforts. 

All the aforementioned aspects of the recent Zika outbreaks highlight the urge for the development of a safe and effective vaccine against Zika. Since 2016, a number of candidates using multiple vaccine platforms have been developed and have shown promising results in preclinical testing. In this review, we discuss the advances in the development of these vaccines. Recent results of phase I clinical trials on Zika vaccine candidates are summarized and discussed. In addition, we address important remaining challenges for the clinical development and future deployment of Zika vaccines.

## 2. Zika Virus Vaccine Development 

**Figure 1:**
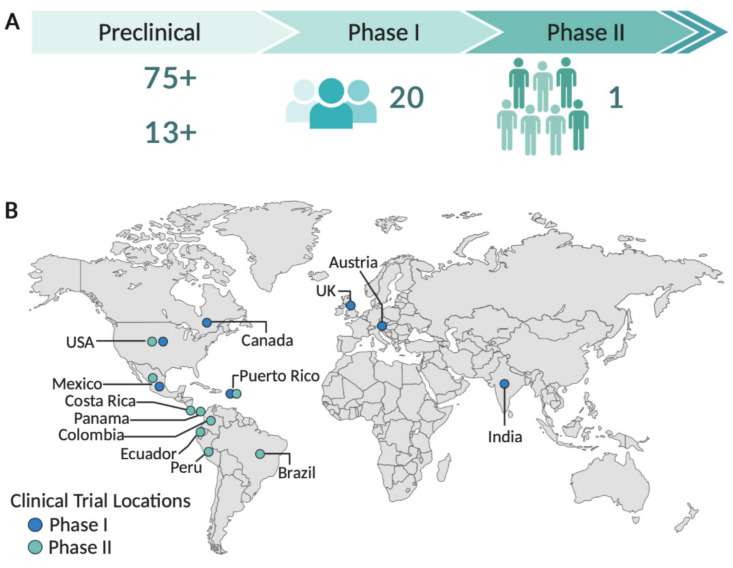
Zika vaccine development. (**A**) Number of Zika vaccine candidates tested in preclinical studies using mice models and non-human primates, and in phase I and II clinical trials in humans; (**B**) phase I and II clinical trial sites. Created with BioRender.com.

The emergence and rapid spread of Zika virus throughout the Americas in 2015 as well as the establishment of causal link between Zika infection and congenital syndrome and neurological disorders triggered unprecedented efforts towards the development of a Zika vaccine. Over the past 5 years, multiple vaccine platform technologies have been used in the design of vaccine prototypes (Figure 1), and 20 are currently in clinical evaluation in humans (Table 1). The accelerated development of multiple Zika vaccine candidates have benefited from over three decades of extensive research on flavivirus immunity, flavivirus vaccine studies, and the quick response of the research community in generating knowledge on Zika biology, pathogenesis, and animal models.

The use of traditional methods, such as the production of empirical, live-attenuated vaccines by repeated passages of virus in cell culture or in animals, or conventional viral inactivation methods have been proven efficacious for the development of multiple flavivirus vaccines. Safe and efficacious vaccines for the prophylaxis of yellow fever virus (YFV), tick-borne encephalitis virus (TBEV) and Japanese encephalitis virus (JEV) have been administered to human populations (reviewed at [66]). The live-attenuated YFV 17D vaccine has been shown to be highly effective and is currently administered to individuals aged 9 months or older, living in or traveling to endemic countries. This vaccine has led to significant reduction in the number of cases and deaths by YFV in the past 80 years. Another licensed, live-attenuated vaccine based on the SA 14-14-2 strain of the JEV has also been extensively used in China and other Asian countries to protect against infections by JEV in children and adults. Inactivated vaccines are also available to prevent JEV and TBEV-induced disease in endemic countries and have been shown to be safe and to engender protection. In addition to these traditional vaccine approaches, a JEV-YFV chimera vaccine developed using innovative molecular biology clone technologies for viral attenuation has been licensed for use in humans. A single dose of this chimera JEV-YFV vaccine has been shown to be safe and to induce sustained antibody responses in clinical trials. In addition, the vast experience in the development of tetravalent DENV vaccines [67] has also contributed to the development of Zika vaccines. There is one DENV vaccine approved for use in several countries (Sanofi-Dengvaxia) that is based on YFV/DENV chimeras. However, the deployment of this vaccine requires confirmation of a previous DENV infection prior to immunization [68]. Other dengue vaccine candidates include a vaccine based on chimeras with an attenuated DENV-2 strain (Takeda-TDV) that have completed a phase III trial, and a vaccine based on a 30-nucleotide deletion in the 3′ untranslated region (UTR) to reduce the efficiency of the viral replication, which is currently concluding its phase III trial in Brazil (Butantan-TDV).

To date, there is no single approved therapeutic or vaccine against Zika, but lessons from these positive stories in flavivirus vaccinology has prompted optimism in the development of Zika vaccines. The WHO vaccine development technology roadmap lays out two priority scenarios for Zika vaccine deployment: epidemic and endemic uses [69]. Despite low levels of transmission during recent years, ZIKV has become endemic in the Americas and the threat of large Zika outbreaks remains. Mass Zika vaccination targeting pregnant women and women of childbearing age populations during outbreaks would likely prevent prenatal Zika infection and thus congenital Zika syndrome cases. In the case of endemic usage, routine Zika immunization of children and adults living in or traveling to endemic countries would allow the establishment of population immunity and aid the prevention of both prenatal Zika infection in pregnant women and other Zika-related neurological complications in adults. 

Numerous Zika vaccine platforms have been developed and tested in preclinical and clinical studies. These vaccines include classical vaccine designs (inactivated virus vaccines and live-attenuated vaccines) and novel vaccine technologies (viral-vectored vaccines and nucleic acid vaccines). Whole, inactivated viruses are prepared by exposing virus particles to chemical agents or heat. Virion then becomes noninfectious and thus unable to cause disease [70]. Live-attenuated vaccine approaches can be developed by different methods to attenuate virions until the virus effectively loses its pathogenic properties. These classical vaccine approaches have been proven to be safe and cost-effective, but they also have some disadvantages. Inactivated vaccines are considered safer than live vaccines, thus potentially offering a higher chance of being used by high-priority groups, such as pregnant women. However, chemical inactivation can alter the molecular structure of antigens and thereby negatively influence vaccine immunogenicity. In addition, inactivated vaccines usually require the use of adjuvants to stimulate robust immune responses. On the other hand, live-attenuated vaccines usually induce a more potent immune response than inactivated vaccines, as they induce innate responses as well as B- and T-cell responses against structural and nonstructural antigens, affording vaccinated individuals with long-term protection. However, live-attenuated vaccines are not administered to individuals with weakened immune systems and their use is also not recommended in pregnant populations unless their benefits outweigh the risks associated with vaccination [70,71]. Platforms such as Virus-like Particles (VLPs) have also been developed as an alternative to classical vaccine designs. VLP-based vaccines are nonreplicating structures composed of viral structural proteins that resemble virions. This vaccine strategy is considered safer than live-attenuated and inactivated virus vaccine approaches since the potential for reversion to virulence and incomplete inactivation are eliminated. Recent preclinical data have shown that VLP-based vaccine candidates elicit strong neutralizing antibody responses in mice [72,73,74]. Specifically, VLPs displaying the third domain (DIII) of the ZIKV envelop (E) protein were able to elicit antibodies that neutralize ZIKV without enhancing DENV infection in vitro [72,73]. If results are confirmed in larger animal models, this vaccine platform might constitute a good candidate for further clinical trials in humans.

Viral-vectored and nucleic acid vaccines are developed based on the coding sequence information of the viral genome alone through genetic engineering techniques [71]. With the aid of recombinant DNA techniques, nucleic acid and viral vectors are engineered to express the antigens of interest. Replication-deficient forms of this vaccine design are safe and relatively stable. Viral-vectored vaccines produce protein antigens endogenously and thus induce potent humoral and cellular immune responses. One disadvantage of this approach is the potential to induce antivector immunity, potentially reducing the immunogenicity of later inoculations using the same viral vector. DNA and mRNA vaccines are based on the delivery of gene coding specific antigens. Following uptake into cells, DNA constructs are transcribed and translated into target proteins by host cells. Constructs of mRNA work in a similar manner, but bypass the transcription step [75]. Nucleic acid vaccine designs can induce potent humoral and cellular responses, especially when the antigens are engineered to target the MHCII processing compartment [76,77], but one limitation of this approach is the limited efficiency of cellular uptake of nucleic acids. Often, nucleic acid vaccines require additional delivery devices to facilitate nucleic acid entry into cells [71,75]. To date, approximately 90 studies have been published reporting data on preclinical testing of Zika vaccine candidates using mice and non-human primate models (Figure 1). Several of these vaccine approaches showed promising results in animal models and advanced to clinical trials in humans.

## 3. Zika Virus Vaccines Candidates in Clinical Trials

As of September 2020, 20 phase I clinical trials have been registered in the clinicaltrials.gov platform (Table 1). Of those, eight phase I clinical trials have their interim or final results published in peer-reviewed journals (Table 2). So far, only one vaccine has advanced to phase II trials. The vast majority of the phase I trials were carried out in nonendemic areas of the United States and Europe. Four phase I clinical trials were conducted in Puerto Rico, an area with active Zika virus transmission. Phase II clinical trial sites include endemic and nonendemic areas of Zika transmission, including several sites in Latin America (Figure 1).

### 3.1. DNA Vaccines

Three different Zika DNA vaccines have entered phase I clinical trials (Table 1). The first report of a Zika vaccine candidate in phase I clinical trial was published in 2017 (NCT02809443) [82], only 2 years after the first Zika outbreak in Latin America. This open label clinical trial evaluated the safety and immunogenicity of a synthetic DNA vaccine encoding the pre-membrane (prM) and E proteins of Zika (GLS-5700). GLS-5700 was administered via intradermal injection followed by electroporation, in a prime-boost regimen consisting of three doses of vaccine. The vaccine was tested in healthy, dengue-seronegative adults. Results from this trial showed that the DNA vaccine was well-tolerated and did not elicit any severe adverse effect in the volunteers. All participants developed ZIKV-binding antibody responses. Around 60% of participants developed ZIKV-neutralizing antibody response, but at low titers (1:18 to 1:317) and that did not correlate with vaccine dose. Participants also developed moderate T-cell response after vaccination. Importantly, this immune response was protective in both in vitro and in vivo (adoptive transfer) challenge models [82]. Another phase I trial designed to evaluate safety, tolerability, and immunogenicity of GLS-5700 in dengue-seropositive adults has been completed (NCT02887482), but results are yet to be published.

Results from two other phase I clinical trials assessing safety and immunogenicity of Zika DNA vaccine candidates were jointly published in late 2018 [81]. In these trials, two vaccine formulations were independently tested: one wild-type ZIKV DNA vaccine formulation (VRC 5283) and one chimera JEV/ZIKV DNA vaccine (VRC 5288). Both vaccine constructs express ZIKV E antigen (French Polynesian strain), but in the JEV/ZIKV chimera, the stem and transmembrane regions of the E protein of ZIKV were modified to encode JEV sequences. In addition, in both vaccines the prM was comprised of analogous sequence from JEV to improve particle secretion. No information about previous flavivirus profile of the volunteers was provided in these publications. Different vaccine doses and inoculation methods (intramuscularly using a needle syringe or a needle-free device) were tested (Table 2). Both DNA vaccine constructs were safe and well-tolerated and showed no severe adverse outcomes in healthy adult volunteers. VRC5283 showed the greatest immunogenicity given in split doses via needle-free injection All participants in the VRC5283 trial had detectable ZIKV antibody responses as well as ZIKV-neutralizing antibody, and showed CD4 and CD8 T-cell responses of the greatest magnitude when compared to VRC 5288 [81]. VRC5283 has advanced to an international placebo-controlled phase II trial. The efficacy of a three-dose (0, 4, and 8 weeks) vaccination regimen of VRC5283 administered via needle-free delivery with the Stratis device (NCT03110770) is being assessed in adults and adolescents residing in flavivirus endemic and nonendemic areas.

### 3.2. Purified, Inactivated Virus Vaccines

Four different Zika inactivated vaccines have entered phase I clinical trials (Table 1): two of those have had their results recently published and the other two have completed phase I trial, but results are yet to be announced. In 2018, Modjarrad and colleagues [80] published the interim, aggregated results of three phase I clinical trials of a purified, formalin-inactivated Zika vaccine (ZPIV) (NCT02963909, NCT02952833, and NCT02937233). In these trials, healthy adults randomly received 5 µg of either ZIPV with alum adjuvant or saline placebo intramuscularly at weeks 0 and 4. All participants in these trials were initially reported to be flavivirus-naïve, but some participants were later found to be flavivirus positive in a post hoc analysis. ZPIV vaccine caused only minor to moderate reactogenicity. By day 57 of follow-up, more than 90% of the participants seroconverted to Zika neutralizing antibodies. However, 15% of those have geometric mean titers (GMT) lower than 100. Adoptive transfer of purified IgG collected at day 57 of follow-up provided robust protection against viremia in ZIKV-challenged mice [80].

In 2020, Stephenson and colleagues [79] published additional results for this vaccine in a trial comparing different doses and vaccination schedules of the ZPIV vaccine (NCT02937233). The focus of this trial was on vaccination schedule. In addition to testing a two-dose ZPIV schedule at weeks 0 and 4, which is a standard regimen for purified inactivated flavivirus vaccines, authors also examined the safety and immunogenicity of a single-dose regimen and an accelerated two-dose regimen with ZPIV given at weeks 0 and 2. Volunteers have no known history of previous flavivirus infection or vaccination. High antibody titers were observed when using the standard dose regimen. However, at week 28, a positive response was observed in only 13% of the participants who received ZPIV via the standard regimen (*n* = 8) and in none of the participants who received ZPIV via the accelerated (*n* = 7) or single-dose (*n* = 10) regimens (Table 2). Importantly, ZIKV-neutralizing antibody levels declined to a GMT of less than 100 by week 16. This short durability of antibody response is probably related to the lack of induction of robust T cell responses. Of note, the accelerated regimen elicited similar peak of ZIKV-neutralizing antibody titers when compared to the standard regimen, but this peak concentration was achieved sooner. However, the antibody response of both regimens was not durable [79]. Additional trials of the ZPIV vaccine have been completed and include testing two doses of ZPIV and a late ZPIV boost (NCT02963909), as well as higher ZPIV doses (NCT02952833) in healthy, flavivirus-naïve adult individuals. Another phase I trial is assessing ZPIV immunogenicity in healthy adults residing in a flavivirus endemic area (NCT03008122) (Table 1).

Recently, Han and colleagues [78] reported the results of a phase I clinical trial on the safety, dose ranging, and immunogenicity (NCT03343626) of a different purified, formalin-inactivated, alum-adjuvated whole Zika virus vaccine candidate (PIZV). So far, this is the first large phase I trial that compared vaccine immunogenicity in flavivirus-naïve versus flavivirus-primed volunteers (Table 2). Authors tested three doses (2, 5, and 10 µg) of this vaccine formulation and all were reported to be safe and well-tolerated in healthy adults with and without preexisting exposure to other flaviviruses throughout 57 weeks of follow-up. A two-dose regimen of the vaccine administered 28 days apart elicited a robust, dose-dependent Zika virus-neutralizing antibody response in 100% of the flavivirus-naïve participants. Neutralizing antibody GMT among flavivirus-naïve individuals remained >3000 in the 10 µg vaccine group by study week 57. A similar dose-dependent neutralizing antibody response was also noted among flavivirus-primed volunteers, but GMTs did not increase following second dose and tended to be lower than those in the flavivirus-naïve group [78].

### 3.3. mRNA Vaccines

Two mRNA vaccine candidates, named mRNA-1325 (NCT03014089) and mRNA-1893 (NCT04064905), have entered phase I clinical trials (Table 1). These vaccine constructs incorporate prM/E genes of ZIKV, but differ with respect to the signal peptide at the amino terminus of prM. mRNA-1325 expresses the signal sequence from human IgE upstream of prM [83]. Both vaccine constructs were immunogenic and protected against Zika infection in multiple mouse models. Interim phase I data for mRNA-1893 were press released in April 2020 by the sponsors (ModernaTX Inc., Cambridge, MA, United States) [84]. A two-dose (10 or 30 µg) vaccination schedule of mRNA-1893 given 28 days apart was well-tolerated in healthy adults with and without preexisting exposure to other flaviviruses. No serious vaccine-related adverse events were reported by volunteers. Both 10 and 30 µg dose levels were immunogenic. All flavivirus-seronegative individuals seroconverted to Zika-neutralizing antibodies in the 30 µg dose group after the second vaccination. In the group consisting of flavivirus-primed participants, 75% achieved a four-fold boost in preexisting neutralizing titers following a second vaccination at the 30 µg dose. These results are yet to be published in peer-reviewed journals.

### 3.4. Live Attenuated Vaccines

Several strategies to develop a live-attenuated Zika vaccine have been employed, but despite promising preclinical data only one live-attenuated Zika vaccine candidate has entered phase I clinical trial so far (NCT03611946). Live-attenuated vaccine approaches include chimeric flavivirus constructs expressing ZIKV prM/E in the genetic background of YFV, JEV, or DENV [85,86,87,88] and nucleotide deletion in the 3′ UTR [89,90]. These vaccines have been proved immunogenic and protective in both mice and non-human primate models [85,86,87,88,89,90]. The live-attenuated chimeric rZIKV/D4D30-713 vaccine has completed phase I clinical trial in healthy, flavivirus-naïve adults (NCT03611946). This vaccine expresses ZIKV prM-E proteins in the genomic backbone of DENV-4 and encodes a 30-nucleotide deletion in the 3′ UTR to attenuate viral replication. Preclinical and phase I data on this vaccine have not yet been reported.

### 3.5. Viral-Vectored Vaccines

Different viral-vectored vaccines expressing ZIKV prM/E proteins have entered phase I clinical trials (Table 1). These viral vectors include adenovirus and measles vaccine virus as the delivery platform for ZIKV antigens. Preclinical evaluation of a recombinant measles Schwarz vaccine (MV-ZIKA) vector expressing ZIKV prM-E reduced plasma viremia and viral load in distinct organs and in the placenta, preventing fetal infection [91]. MV-ZIKA has completed phase I clinical trial, but results are yet to be published (NCT02996890). Another similar measles-based vaccine (MV-ZIKV-RSP) has entered phase I trials and the study is currently recruiting participants in Austria (NCT04033068). A replication-deficient vaccine candidate based on chimpanzee adenovirus (ChAdOx1) encoding ZIKV prM-E has also proved protective against ZIKV challenge in mice. This vaccine elicited long-lasting anti-envelope immunity in mice with no evidence of enhancement of dengue virus in vitro [92]. ChAdOx1 has entered two phase I clinical trials to determine its safety and immunogenicity in healthy adults when administered as a standalone vaccine or coadministered with a Chikungunya virus (CHIKV) vaccine (NCT04015648 and NCT04440774). Immunization with a replication-incompetent human adenovirus type 26 (AD26)-vectored vaccine encoding ZIKV M and E proteins (Ad26.ZIKV.001) induced robust ZIKV binding and neutralizing antibody responses as well as cellular responses in mice and non-human primates during preclinical evaluation [93]. A single dose of Ad26.ZIKV.001 provided full protection against Zika challenge in several mice models and non-human primates. This vaccine has advanced to phase I clinical trial (NCT03356561).

## 4. Challenges in Late Stage Development of Zika Vaccines and Future Perspectives

Despite the fast-paced start and promising phase I clinical trial results, several challenges for the late development of ZIKV vaccine candidates still remain. Further development of Zika vaccines will require efficacy studies in phase III trials and these trials face major challenges. Typical flavivirus vaccine efficacy trials require the detection of a large number of virologically confirmed cases and comparison of the number of cases or the viremia levels between immunized and control groups. However, in order to compensate for the high proportion of asymptomatic/unapparent ZIKV infections, these studies would need to enroll a relatively large number of volunteers as compared to dengue vaccine studies. In addition, following the explosive 2015/2016 outbreaks, ZIKV transmission has been dramatically reduced. Thus, in the context of low level of transmission rates, the number of volunteers required would increase even further, making the assessment of clinical vaccine efficacy even more difficult [94]. In addition, the ultimate goal of a Zika vaccine is the prevention of congenital disease; measuring this effect directly in a phase III trial would be extremely difficult and costly. Therefore, it is crucial to better characterize the pathogenic mechanisms involved in Zika congenital disease in order to identify and validate correlates of immunity to be employed in the evaluation of the efficacy of vaccine candidates. It is also critical to take into consideration the challenge of developing a business model to attract enough investment in order to continue developing a Zika vaccine and, subsequently, maintain a sustained production of it. As with several other newly emerging pathogens that have threatened human health on a global scale, efforts for vaccine development usually decline when the epidemic wanes. Robust public and private funding must continue to be provided if we are to support phase III clinical trials of Zika vaccine candidates. One possible strategy to reduce the cost of phase III trials would be to have the trials and study sites pre-planned and ready to start on short notice when a new outbreak emerges. 

Substantial concern also exists regarding the interactions between preexisting flavivirus immunity and the potential for vaccine-mediated ADE of disease. There has been a growing body of work supporting the idea that the balance between the concentration of cross-reactive antibodies at the time of infection and the interval between two infections are important factors influencing protection against or enhancement of flavivirus disease. In humans, enhancement of dengue disease has been shown to occur within a narrow range of preexisting DENV antibody concentration [95]. In Zika, high titers of preexisting DENV antibodies have been associated with protection against subsequent ZIKV infections in humans [58]. Recent evidence from experimental [59] and epidemiological [57] studies also suggests that low Zika antibody levels may affect clinical outcome of future dengue disease in humans by increasing the risk for severe dengue. How immunity to Zika vaccine candidates might shape future flavivirus epidemics needs to be addressed. Regardless of the platform, most candidates in phase I trials have focused on vaccine strategies targeting antigens (e.g., E and/or prM) that are known to elicit cross-reactive antibody responses. Ideal vaccine candidates should elicit a threshold of cross-reactive antibody concentration that is sufficient to effectively neutralize virus infection, thus providing protection and avoiding vaccine-mediated ADE effects. Of note, most phase I trials have tested the safety and immunogenicity of their vaccine candidates in naïve and previously exposed flavivirus individuals. Long-term analysis of the persistence of vaccine-elicited antibody response and interactions with other flavivirus will be critical to address potential risk for vaccine-mediate enhancement of subsequent flavivirus disease in humans. The possibility of vaccine-mediated enhancement of disease would require careful deployment of the eventual Zika vaccine to be performed only in the age groups with high DENV seroprevalence or only in individuals with a confirmed DENV infection.

Finally, an ideal Zika vaccine needs to be safe for at-risk populations, including women of childbearing age, pregnant women, young children, elderly, and immune-compromised individuals. To achieve such a broad population spectrum, multiple vaccine platforms may be required. In addition, a Zika vaccine should not induce Guillain–Barré-like neurological side effects or congenital malformations in fetuses. Most of the Zika vaccine candidates currently in human trials have been shown to protect fetus against ZIKV challenge and prevent neurological, placental, and testis damage from it in mice and non-human primate models, but if this will hold true in humans still needs to be determined in clinical trials.

## 5. Concluding Remarks

A number of Zika vaccine candidates have completed phase I trial and have been shown to be safe, well tolerated, and immunogenic in healthy adult volunteers. These viable Zika vaccine candidates should continue their development. ZIKV has become endemic and new cases of Zika congenital syndrome continue to be reported in endemic areas. It is very likely that we will face another large Zika epidemic in the next 10–15 years as herd immunity decay. Therefore, it is critical for Zika vaccine developers to be ready to activate phase III trials and ramp up vaccine production in case another epidemic emerges. Vaccine developers should also consider coordinating strategies to use Dengue and Zika vaccines to maximize the immunogenicity of both vaccines and to reduce the risks associated with deleterious DENV and ZIKV cross-reactive antibody interactions. Moreover, the coordinated use of both vaccines may also lead to a more attractive business model. 

## Figures and Tables

**Table 1:** Zika vaccine candidates in clinical trials.

Vaccine Platform	Vaccine Name	Developer(s)	Study Description/Current Status	Clinical Trial
Purified Inactivated Vaccines	Zika Virus Purified Inactivated Vaccine (ZPIV)	NIAID, WRAIR, BIDMC	Phase I trial on the safety, reactogenicity and immunogenicity, and comparison of different doses and schedules of ZPIV in healthy, flavivirus-naïve adult individuals—Completed	NCT02937233
			Phase I trial on the safety, reactogenicity and immunogenicity of a higher ZPIV dose in healthy, flavivirus-naïve adult individuals—Completed	NCT02952833
			Phase I trial on the safety, reactogenicity and immunogenicity of two doses of ZPIV and a late ZPIV boost in healthy, flavivirus-naïve adult individuals—Completed	NCT02963909
			Phase I trial on the safety, reactogenicity and immunogenicity of two doses of ZPIV in healthy adults residing in a flavivirus endemic area—Active, not recruiting	NCT03008122
	Purified Inactivated Zika Virus Vaccine (PIZV)	Takeda	Phase I trial on the safety, immunogenicity and dose ranging of PIZV in flavivirus-naïve and primed healthy adults—Active, not recruiting	NCT03343626
	VLA1601	Valneva Austria GmbH, Emergent BioSolutions	Phase I trial on the safety and immunogenicity of two dose levels of the VLA1601 vaccine in healthy, flavivirus-naïve adults—Completed	NCT03425149
	BBV121	Bharat Biotech International Limited	Phase I trial to evaluate two doses of three sequentially escalating cohort of BBV121 in healthy adult dengue seronegative and dengue seropositive volunteers—Completed	NCT04478656
DNA Vaccines	VRC5288 (Zika virus and Japanese encephalitis virus chimera) VRC5283 (wild-type Zika virus)	NIAID, VRC	Phase I trial on the safety and immunogenicity of VRC5283 administered via needle and syringe or needle-free injector in healthy adults—Completed	NCT02996461
			Phase I/Ib trial on the safety, tolerability, and immunogenicity of VRC5288 administered via needle and syringe in healthy adults—Completed	NCT02840487
	VRC5283 (wild-type Zika virus)	NIAID, VRC, Emmes Company, Leidos Biomedical Research, FHI Clinical, PPD	Phase II/IIb trial on the safety, immunogenicity, and efficacy of a three-dose vaccination regimen of the VRC5283 administered via needle-free device in healthy adults and adolescents residing in flavivirus endemic and nonendemic areas—Completed	NCT03110770
	GLS-5700	GeneOne Life Science, Inc., Inovio Pharmaceuticals	Phase I trial, dose-ranging study to evaluate the safety, tolerability, and immunogenicity of GLS-5700 in healthy, dengue-naïve adults—Completed	NCT02809443
			Phase I trial to evaluate the safety, tolerability, and immunogenicity of GLS-5700 in dengue-seropositive adults—Completed	NCT02887482
mRNA Vaccines	mRNA-1893	ModernaTX, Inc., Biomedical Advanced Research and Development Authority	Phase I dose-ranging study to evaluate the safety, tolerability, and immunogenicity of mRNA-1893 in healthy flavivirus-seropositive and seronegative adults—Active, not recruiting	NCT04064905
	mRNA 1325		Phase I dose-ranging study to evaluate the safety and immunogenicity of mRNA 1325 in healthy adults in a nonendemic Zika region—Completed	NCT03014089
Live-Attenuated Vaccines	rZIKV/D4Δ30-713	NIAID	Phase I trial to evaluate the safety, reactogenicity, and immunogenicity of a single dose of the rZIKV/D4Δ30-713 vaccine in healthy, flavivirus-naïve adults—Completed	NCT03611946
Viral Vectored Vaccines	MV-ZIKA-RSP (Measles virus-based)	Themis Bioscience GmbH	Phase I trial comparing different dose levels of the MV-ZIKA-RSP vaccine to evaluate the safety, tolerability, and immunogenicity in healthy adults—Recruiting	NCT04033068
	MV-ZIKA		Phase I dose-finding study to evaluate the optimal dose of MV-ZIKA and to asses immunogenicity, safety, and tolerability in healthy adult volunteers—Completed	NCT02996890
	ChAdOx1 Zika (Chimpanzee Adenovirus-based)	University of Oxford	Phase I trial to determine the safety and immunogenicity of ChAdOx1 Zika as a standalone vaccine or coadministered with a CHIKV vaccine (ChAdOx1 Chik) in healthy adults—Recruiting	NCT04015648
			Phase Ib trial to evaluate the safety and immunogenicity of the ChAdOx1 Zika vaccine as a standalone vaccine or coadministered with ChAdOx1 Chik in healthy adults in Mexico—Not yet recruiting	NCT04440774
	Ad26.ZIKV.001 (Adenovirus serotype 26-based)	Janssen Vaccines and Prevention B.V.	Phase I trial to evaluate the safety, reactogenicity and immunogenicity of Ad26.ZIKV.001 in healthy adult volunteers—Completed	NCT03356561

NIAID, National Institute of Allergy and Infectious Diseases; WRAIR, Walter Reed Army Institute of Research (WRAIR), BIDMC, Beth Israel Deaconess Medical Center; VRC, Vaccine Research Center; ZIKV, Zika Virus; CHIKV, Chikungunya virus.

**Table 2:** Zika vaccine candidates with results of their phase I clinical trials published in peer-reviewed journals.

Vaccine Platform	Name/Sponsor	Antigen	Regimen (Dosage, Intervals, Route)	Study Design	Subject Characteristics	Clinical Trial/Reference
Purified Inactivated Virus	PIZV or TAK-426 Takeda Pharmaceuticals	Whole Virus, PRVABC59 strain/Aluminum hydroxide adjuvant	2, 5 or 10 μg 0–4 week Deltoid, IM	Multicenter, randomized, observer-blind, placebo-controlled	Healthy adults, aged 18–49 years *n* = 271 participants Two groups: flavivirus-naïve (*n* = 125) and flavivirus-primed (*n* = 146)	NCT03343626 [78]
	ZPIV NIAID, WRAIR, BIDMC	Whole Virus, PRVABC59 strain/Aluminum hydroxide adjuvant	5 μg Three intervals: 0–2; 0–4 week; or single dose Deltoid, IM	Single-center, randomized, double-blind, placebo-controlled	Healthy adults, aged 18–50 years *n* = 36 participants No known history of previous flavivirus infection or vaccination	NCT02937233 [79]
			5 μg 0–4 week Deltoid, IM	Single-center (three independent trials), randomized, double-blind, placebo-controlled	Healthy adults, aged 18–49 years *n* = 67 participants No known history of previous flavivirus infection or vaccination	NCT02963909 NCT02952833 NCT02937233 [80]
DNA Vaccine	VRC5288 and VRC5283 NIAID/VRC	prM and E VRC5288 (ZIKV and JEV chimera) VRC5283 (wild-type ZIKV)	4 mg VRC5288 0–8, 0–12; 0–4–8; or 0–4–20 week Deltoid, IM (needle and syringe) VRC5283 0–4–8 week Deltoid, IM (Single or Split-dose needle and syringe or needle-free with Stratis device)	Two independent trials (VRC 319, multicenter; and VRC320, single-center), randomized, open-label	VRC 319 (VRC5288 vaccine) Healthy adults, aged 18–35 years *n* = 80 participants VRC 320 (VRC5283 vaccine) Healthy adults, aged 18–50 years *n* = 45 participants	NCT02840487 NCT02996461 [81]
	GLS-5700 GeneOne Life Science, Inc., Inovio Pharmaceuticals	prM and E	1 or 2 mg 0–4–12 week Deltoid, IM followed by electroporation (CELLECTRA-3P)	Multicenter, nonrandomized, open-label, placebo-controlled	Healthy adults, aged 18–65 years *n* = 40 participants Dengue-seronegative	NCT02809443 [82]

NIAID, National Institute of Allergy and Infectious Diseases; WRAIR, Walter Reed Army Institute of Research (WRAIR), BIDMC, Beth Israel Deaconess Medical Center; VRC, Vaccine Research Center; ZIKV, Zika Virus; JEV, Japanese Encephalitis Virus; IM, intramuscular.
